# HIV-1 Viremia and Cancer Risk in 2.8 Million People: The South African HIV Cancer Match Study

**DOI:** 10.1093/cid/ciae652

**Published:** 2024-12-30

**Authors:** Yann Ruffieux, Judith Mwansa-Kambafwile, Carole Metekoua, Tinashe Tombe-Nyahuma, Julia Bohlius, Mazvita Muchengeti, Matthias Egger, Eliane Rohner

**Affiliations:** Institute of Social and Preventive Medicine, University of Bern, Bern, Switzerland; National Cancer Registry, National Health Laboratory Service, Johannesburg, South Africa; School of Public Health, University of Witwatersrand, Johannesburg, South Africa; Institute of Social and Preventive Medicine, University of Bern, Bern, Switzerland; National Cancer Registry, National Health Laboratory Service, Johannesburg, South Africa; Graduate School for Health Sciences, University of Bern, Bern, Switzerland; National Cancer Registry, National Health Laboratory Service, Johannesburg, South Africa; Institute of Social and Preventive Medicine, University of Bern, Bern, Switzerland; Swiss Tropical and Public Health Institute, Allschwil, Switzerland; University of Basel, Basel, Switzerland; National Cancer Registry, National Health Laboratory Service, Johannesburg, South Africa; School of Public Health, University of Witwatersrand, Johannesburg, South Africa; South African DSI-NRF Centre of Excellence in Epidemiological Modelling and Analysis (SACEMA), Stellenbosch University, Stellenbosch, South Africa; Institute of Social and Preventive Medicine, University of Bern, Bern, Switzerland; Population Health Sciences, Bristol Medical School, University of Bristol, Bristol, United Kingdom; Centre for Infectious Disease Epidemiology and Research (CIDER), School of Public Health, University of Cape Town, Cape Town, South Africa; Institute of Social and Preventive Medicine, University of Bern, Bern, Switzerland

**Keywords:** HIV-1 viremia, cancer, incidence, South Africa, linkage study

## Abstract

**Background:**

Most research on human immunodeficiency virus-1 (HIV-1) viremia and cancer risk is from high-income countries. We evaluated the association between HIV-1 viremia and the risk of various cancer types among people with HIV (PWH) in South Africa.

**Methods:**

We analyzed data from the South African HIV Cancer Match study, based on laboratory measurements from the National Health Laboratory Service and cancer records from the National Cancer Registry from 2004 to 2014. Using Cox proportional hazards models, we estimated hazard ratios (HR) for cancer incidence per unit increase in time-updated Log10 HIV-1 RNA viral load copies/mL. We created partially adjusted (sex, age, calendar year) and fully adjusted models (additionally including time-updated CD4 count).

**Results:**

We included 2 770 200 PWH with 10 175 incident cancers; most common were cervical cancer (N = 2481), Kaposi sarcoma (N = 1902), breast cancer (N = 1063), and non-Hodgkin lymphoma (N = 863). Hazard ratios for the association of HIV-1 viremia and cancer risk changed after partial and full adjustment and were generally attenuated for infection-related cancers but tended to increase for infection-unrelated cancers. In the fully adjusted model, HIV-1 viremia was associated with an increased risk of Kaposi sarcoma (HR per unit increase in Log10 HIV-1 RNA viral load: 1.38; 95% confidence interval [CI], 1.35–1.42), leukemia (HR: 1.28; 95% CI, 1.13–1.45), non-Hodgkin lymphoma (HR: 1.24; 95% CI, 1.19–1.29), conjunctival cancer (HR: 1.19; 95% CI, 1.11–1.25), and colorectal cancer (HR: 1.11; 95% CI, 1.02–1.21). Associations with other cancer types were weaker or absent.

**Conclusions:**

Our findings underline the importance of sustained viral suppression for cancer prevention among PWH in South Africa.

## Teaser

In this large cohort of people living with HIV in South Africa, HIV-1 viremia was associated with an increased risk of most infection-related cancers and some infection-unrelated cancers both before and after adjusting for CD4 cell count.

In 1996, the International Agency for Research on Cancer categorized human immunodeficiency virus-1 (HIV-1) as carcinogenic in humans [1]. HIV-1–induced cellular immunodeficiency with low CD4 cell counts is strongly associated with several infection-related and some infection-unrelated cancers among people with HIV (PWH) [2–4]. Furthermore, independently of immunodeficiency, HIV-1 replication may increase cancer risk through chronic inflammation, accelerated immunosenescence, and direct pro-oncogenic effects [5–7]. It is well documented that prolonged HIV-1 viremia is associated with an increased risk of Kaposi sarcoma and non-Hodgkin lymphoma [2, 3, 8]. A population-based cohort study from the United States concluded that HIV-1 viremia was also associated with higher rates of non-AIDS–defining cancers [9]. Specifically, HIV-1 viremia may promote the development of anal cancer, Hodgkin lymphoma, and several infection-unrelated cancer types [10, 11].

The scale-up of combination antiretroviral therapy (ART) has decreased the risk of certain cancers among PWH [6, 12]. However, US veterans with incomplete virological suppression of HIV-1 or even those with long-term suppression, remained at higher cancer risk than HIV-negative individuals [11]. Moreover, another US-based study found that non-Hodgkin lymphoma rates were higher among PWH with low-level viremia than those with suppressed viral loads [8]. In contrast, other studies did not find a clear association between HIV-1 viremia after ART initiation and cancer risk [13, 14].

To date, most research of HIV-1 viremia and cancer risk has focused on high-income countries. Studies from sub-Saharan Africa, where most PWH live, are scarce. The South African HIV Cancer Match (SAM) study is a nationwide cohort of PWH in South Africa covering the years 2004–2014 [15]. The SAM study provides a unique opportunity to examine how HIV-1 viremia is linked to the risk of developing cancer in this region, while accounting for immunodeficiency and other factors.

## METHODS

### Study Design and Setting

Described in detail elsewhere [15], the SAM study is based on a probabilistic linkage of records from the National Health Laboratory Service (NHLS) and the National Cancer Registry (NCR) from 2004 to 2014. The NHLS serves the public sector through more than 260 laboratories across all provinces. The NCR collects data on pathologically confirmed cancer diagnoses from public and private laboratories throughout South Africa [16]. We first created a virtual cohort of PWH by identifying HIV-related laboratory records (positive HIV-1 tests, CD4 cell counts and percentages, and HIV-1 RNA viral loads) most likely belonging to the same individual. Then we linked PWH to cancer diagnoses from the NCR. The NHLS and NCR records provided information on a person's sex and age, but not on their ART uptake. During the study period, which preceded the ART “treat all” era, CD4 count and HIV-1 RNA viral load testing were essential components of HIV care in South Africa. HIV-1 RNA viral load testing was initially recommended at ART initiation and every 6 months thereafter. However, the 2010 guidelines removed the requirement for a viral load test at ART initiation [17]. Additionally, although viral load testing was recommended starting in 2004, resource constraints often limited its availability in many public healthcare facilities. Between 2004 and 2014, ART coverage in South Africa increased from 1% to 44% [18]. We obtained ethical approval from the Human Research Ethics Committee of the University of the Witwatersrand, Johannesburg, South Africa, and the Cantonal Ethics committee of Bern, Switzerland.

### Inclusion Criteria and Definitions

We included PWH who had HIV-related laboratory records on different days. At least 1 record had to be an HIV-1 RNA viral load measurement, accompanied by a CD4 cell count taken on or before the day of the first HIV-1 RNA viral load measurement. We defined the time of the first HIV-1 RNA viral load measurement as baseline. We excluded individuals with missing age or sex and individuals aged <18 years at baseline. We excluded individuals diagnosed with a given cancer type before their baseline visit from the analysis of that cancer. An individual's time-at-risk started at their baseline visit and ended 6 months after their last HIV-1 RNA viral load measurement, on the database closing date (1 January 2015), or the diagnosis date of the cancer under consideration, whichever came first.

We identified cancer types based on the International Classification of Diseases for Oncology, 3rd Edition. We distinguished between infection-related and infection-unrelated cancers (Supplementary Table 1). Among infection-related cancers we analyzed Kaposi sarcoma, cervical cancer, other human papillomavirus (HPV)-related cancers, non-Hodgkin lymphoma, Hodgkin lymphoma, and conjunctival cancer. The infection-unrelated cancers included leukemia, breast, colorectal, lung, esophageal, and prostate cancer. We excluded basal cell carcinoma (morphology codes 8090–8110) and squamous cell carcinoma of the skin (topography codes C44.0-9 with morphology codes 8050–8084). We defined viral suppression as an HIV-1 RNA viral load <1000 copies/mL. The most common lower detection limit for HIV-1 RNA viral load was 20 copies/mL. We therefore set all viral load measurements below a detection limit to 10 copies/mL.

### Statistical Analysis

We calculated descriptive statistics for PWH who developed each cancer type. We quantified the association between HIV-1 viremia and cancer rates using hazard ratios (HRs) from Cox proportional hazards models. HIV-1 RNA viral load was our main exposure measure but we also considered the percentage time virologically suppressed and cumulative viral load (Supplementary Table 2). We updated HIV-1 RNA viral load and CD4 cell count at each new measurement, carrying the value forward until the next measurement or censoring. The relationship between Log10 HIV-1 RNA viral load and cancer risk was modelled as linear or nonlinear with a penalized spline transformation. We considered age, sex, and calendar year as potential confounders of the effect of HIV-1 viremia on cancer risk, whereas CD4 cell count may act as a potential mediator. We obtained HRs from unadjusted models, from partially adjusted models accounting for age (18–29, 30–39, 40–49, 50–69, ≥ 70 years), sex, and calendar year (2004–2007, 2008–2011, 2012–2014), and from fully adjusted models additionally accounting for CD4 cell count (0–99, 100–199, 200–349, 350–499, ≥ 500 cells/µL). All variables were time-updated. When computing cumulative HIV-1 RNA viral load, we assumed viral load levels progressed linearly between successive measurements. We lagged the CD4 cell count by 6 months where possible, as cancer development may impact an individual's immunodeficiency level (reverse causality). If no CD4 cell count was available 6 or more months before the first viral load measurement, we used the earliest available CD4 count. We performed sensitivity analyses wherein we: (1) included individuals with no CD4 count at or before baseline, adding a category for missing information, (2) excluded time-at-risk with no 6-month lagged CD4 count, and (3) excluded individuals who ever had a CD4 count <200 cells/µL. Data management and analyses were performed in R version 4.2.3 (R Foundation for Statistical Computing, Vienna, Austria).

## RESULTS

We identified 5 248 648 PWH with laboratory records on separate days. We excluded 1 865 123 (35.5%) individuals because they had no viral load measurement and another 363 391 because no CD4 cell count was available at or before baseline. We also excluded 249 934 PWH for reasons detailed in Supplementary Figure 1. Thus, we included 2 770 200 PWH with 5 532 262 years-at-risk in total and a median of 1.36 years (interquartile range [IQR] 0.50–2.89). The population size and years-at-risk were slightly higher in the analyses of specific cancers.

### Characteristics of Study Population

More than two-thirds of the included PWH were women (n = 1 909 093; 68.9%), and the median baseline age was 36 years (IQR 30–43). The median baseline CD4 count was 278 cells/µL (IQR 158–429) and the median baseline HIV-1 RNA viral load was 150 copies/mL (IQR 10–6800). A total of 1 892376 individuals (68.3%) were virally suppressed (<1000 copies/mL) at baseline. The median number of HIV-1 RNA viral load measurements per individual was 2 (IQR 1–4) for a total of 7 761 185. Incident cancers were diagnosed in 10 175 PWH, including 6529 with an infection-related cancer, 3281 with an infection-unrelated cancer, and 522 with an ill-defined cancer or unknown primary site. The most common cancers were cervical cancer (N = 2481), Kaposi sarcoma (N = 1902), breast cancer (N = 1063), and non-Hodgkin lymphoma (N = 863).

Among PWH with infection-related cancers, the median age at baseline ranged from 35.0 years (Kaposi sarcoma) to 41.1 years (cervical cancer) (Table 1). The median baseline HIV-1 RNA viral load ranged from 399 copies/mL (cervical and other HPV-related cancers, Hodgkin lymphoma) to 26 000 copies/mL (Kaposi sarcoma). The median age at baseline among PWH with infection-unrelated cancers ranged from 36.3 years (leukemia) to 57.2 years (prostate cancer). Median baseline viral load was <1000 copies/mL for all infection-unrelated cancers (Table 2).

**Table 1.** Baseline Characteristics of Individuals Diagnosed With an Infection-related Cancer

	Kaposi Sarcoma	Non-Hodgkin Lymphoma	Cervical Cancer	Other HPV-related Cancers (Anal, Head and Neck, Penile, Vaginal, Vulvar Cancer)	Hodgkin Lymphoma	Conjunctival Cancer	Any Infection-related Cancer
	N (%)	N (%)	N (%)	N (%)	N (%)	N (%)	N (%)
	1902	863	2481	441	212	401	6529
Female	988 (51.9)	476 (55.2)	2481 (100.0)	311 (70.5)	108 (50.9)	265 (66.1)	4698 (72.0)
Median age [IQR]	35.0 [29.9, 41.6]	39.0 [33.1, 45.6]	41.1 [35.1, 47.8]	37.9 [31.5, 45.6]	35.4 [30.5, 42.1]	36.9 [33.1, 43.0]	38.4 [32.7, 45.6]
Calendar period							
2004–2007	574 (30.2)	206 (23.9)	643 (25.9)	128 (29.0)	56 (26.4)	102 (25.4)	1765 (27.0)
2008–2011	907 (47.7)	427 (49.5)	1202 (48.4)	198 (44.9)	104 (49.1)	202 (50.4)	3150 (48.2)
2012–2014	421 (22.1)	230 (26.7)	636 (25.6)	115 (26.1)	52 (24.5)	97 (24.2)	1614 (24.7)
Median CD4 cell count (cells/μL) [IQR]	150 [67, 262]	179 [92, 279]	226 [128, 368]	203 [118, 340]	215 [125, 344]	133 [73, 231]	188 [99, 318]
HIV-1 RNA viral load (copies/mL)^a^							
10–99	395 (20.8)	288 (33.4)	977 (39.4)	165 (37.4)	84 (39.6)	119 (29.7)	2128 (32.6)
100–999	249 (13.1)	142 (16.5)	484 (19.5)	89 (20.2)	46 (21.7)	51 (12.7)	1103 (16.9)
1000–9999	167 (8.8)	56 (6.5)	250 (10.1)	43 (9.8)	24 (11.3)	44 (11.0)	601 (9.2)
10 000–99 999	449 (23.6)	143 (16.6)	429 (17.3)	68 (15.4)	34 (16.0)	69 (17.2)	1225 (18.8)
≥100 000	642 (33.8)	234 (27.1)	341 (13.7)	76 (17.2)	24 (11.3)	118 (29.4)	1472 (22.5)
Median HIV-1 RNA viral load (copies/mL) [IQR]^a^	26 000 [328, 184405]	1117 [24, 110000]	399 [10, 24000]	399 [10, 36000]	399 [19, 13000]	6100 [49, 150000]	1200 [10, 80000]

Abbreviations: HIV-1, human immunodeficiency virus type 1; HPV, human papillomavirus; IQR, interquartile range.

^a^HIV-1 RNA viral loads below the detection limit or ≤20 copies/mL were set to 10 copies/mL.

**Table 2.** Baseline Characteristics of Individuals Diagnosed With an Infection-Unrelated Cancer

	Breast Cancer	Colorectal Cancer	Esophageal Cancer	Leukemia	Lung Cancer	Prostate Cancer	Any Infection-Unrelated Cancer
	N (%)	N (%)	N (%)	N (%)	N (%)	N (%)	N (%)
	1063	248	232	112	293	307	3281
Female	1042 (98.0)	132 (53.2)	107 (46.1)	60 (53.6)	77 (26.3)	0	2026 (61.7)
Median age [IQR]	43.7 [37.4, 51.1]	48.2 [40.1, 56.6]	50.6 [45.0, 57.0]	36.3 [31.8, 46.3]	51.3 [46.0, 56.3]	57.2 [51.9, 62.4]	47.3 [39.0, 54.7]
Calendar period							
2004–2007	273 (25.7)	47 (19.0)	59 (25.4)	28 (25.0)	83 (28.3)	77 (25.1)	861 (26.2)
2008–2011	539 (50.7)	131 (52.8)	104 (44.8)	64 (57.1)	133 (45.4)	150 (48.9)	1638 (49.9)
2012–2014	251 (23.6)	70 (28.2)	69 (29.7)	20 (17.9)	77 (26.3)	80 (26.1)	782 (23.8)
Median CD4 cell count (cells/μL) [IQR]	230 [142, 381]	235 [138, 369]	216 [135, 356]	271 [156, 461]	229 [114, 350]	224 [125, 376]	224 [132, 362]
HIV-1 RNA viral load (copies/mL)^a^							
10–99	463 (43.6)	90 (36.3)	109 (47.0)	48 (42.9)	118 (40.3)	135 (44.0)	1329 (40.5)
100–999	216 (20.3)	57 (23.0)	48 (20.7)	18 (16.1)	54 (18.4)	62 (20.2)	652 (19.9)
1000–9999	90 (8.5)	21 (8.5)	12 (5.2)	9 (8.0)	31 (10.6)	23 (7.5)	295 (9.0)
10 000–99 999	150 (14.1)	43 (17.3)	32 (13.8)	17 (15.2)	31 (10.6)	40 (13.0)	502 (15.3)
≥100 000	144 (13.5)	37 (14.9)	31 (13.4)	20 (17.9)	59 (20.1)	47 (15.3)	503 (15.3)
Median HIV-1 RNA viral load (copies/mL) [IQR]^a^	340 [10, 17 000]	399 [10, 26 544]	205 [10, 17 250]	315 [10, 25 881]	399 [10, 33 000]	320 [10, 18 500]	399 [10, 25 173]

^a^HIV-1 RNA viral loads below the detection limit or ≤20 copies/mL were set to 10 copies/mL.

### HIV-1 Viremia and Cancer Risk

**Figure 1.**
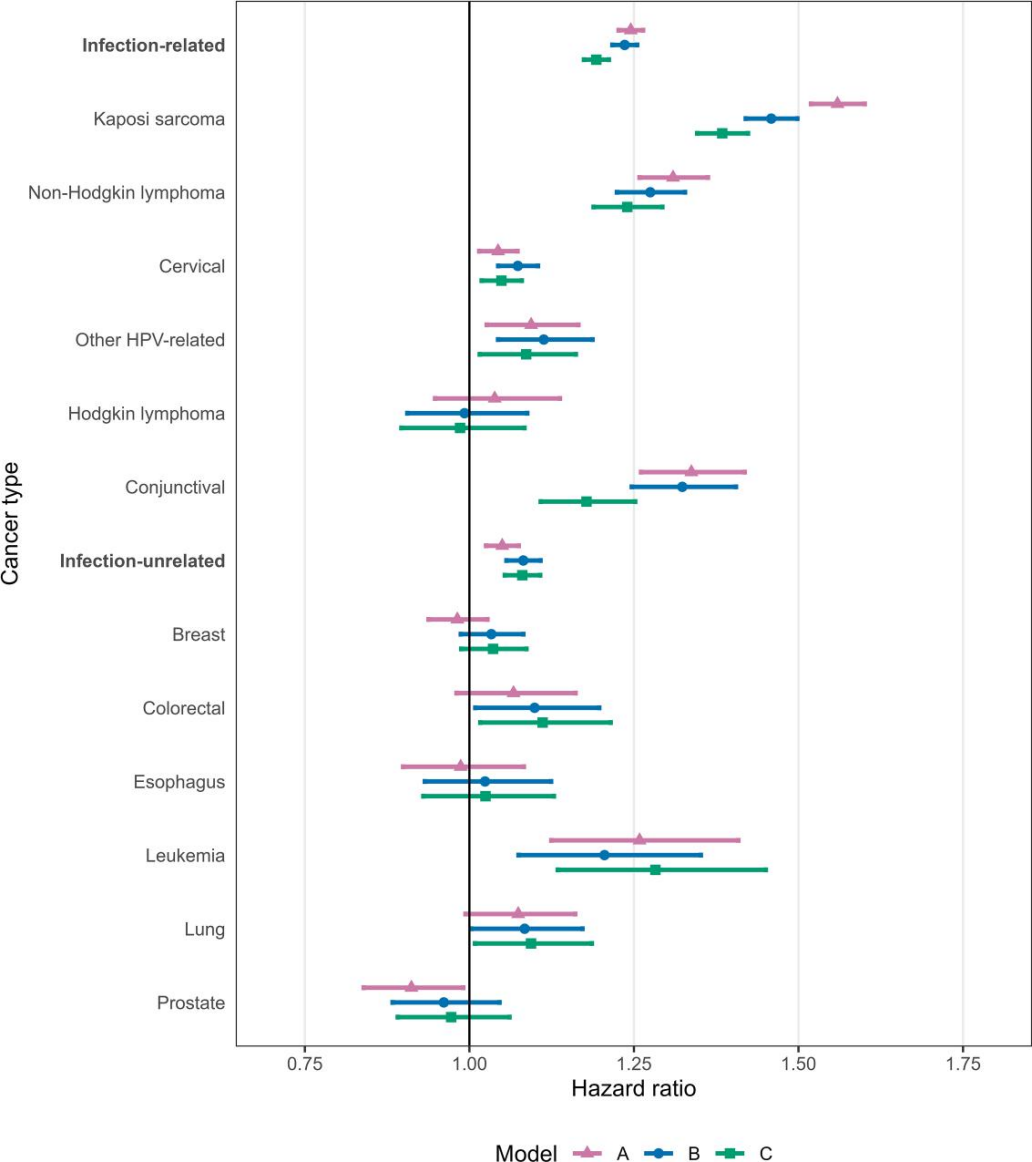
Hazard ratios and 95% confidence intervals for incident cancer per unit increase in Log10 HIV-1 RNA viral load copies/mL. Unadjusted models (*A*), models adjusted for sex, age, calendar year (*B*), and models adjusted for sex, age, calendar year, and CD4 cell count (lagged by 6 months) (*C*). All variables were time-updated. Abbreviation: HPV, human papillomavirus.

HIV-1 viremia was associated with an increased risk of all infection-related cancers except Hodgkin lymphoma and some infection-unrelated cancers (colorectal cancer, leukemia, and lung cancer) (Figure 1). Hazard ratios per unit increase in Log10 HIV-1 RNA viral load copies/mL changed after partial and full adjustment: they were generally attenuated for infection-related cancers and tended to increase for infection-unrelated cancers. The largest change after including CD4 count in the model was observed for Kaposi sarcoma and conjunctival cancer. For Kaposi sarcoma, the unadjusted HR was 1.56 (95% confidence interval [CI],1.52–1.60), decreasing to 1.46 (95% CI, 1.42–1.50) after adjusting for age, sex, and calendar year, and to 1.38 (95% CI, 1.35–1.42) after further adjusting for CD4 count. For conjunctival cancer, the HR decreased from 1.34 (95% CI, 1.26–1.42) in the unadjusted to 1.18 (95% CI, 1.11–1.25) in the fully adjusted model. In contrast, for breast cancer, the HR increased from 0.98 (95% CI, 0.94–1.03) in the unadjusted to 1.04 (95% CI, 0.99–1.09) in the fully adjusted model. Similar trends were observed for the other infection-unrelated cancers. The 3 measures of HIV-1 viremia (HIV-1 RNA viral load, percentage time virally suppressed, cumulative HIV-1 RNA viral load) yielded similar results (Supplementary Tables 3–8).

Our results were robust to changes in the inclusion criteria. Expanding the study population to include 308 341 individuals with no CD4 cell count at or before baseline did not change the results substantially (Supplementary Tables 9–10). Excluding time-at-risk with no available 6-month lagged CD4 cell count (9% of overall time-at-risk) did not lead to noticeably different results (Supplementary Tables 11–12). Excluding PWH who had CD4 counts <200 cells/µL generally strengthened the association between HIV-1 viremia and cancer risk, particularly for infection-related cancers (Supplementary Tables 13–14).

**Figure 2.**
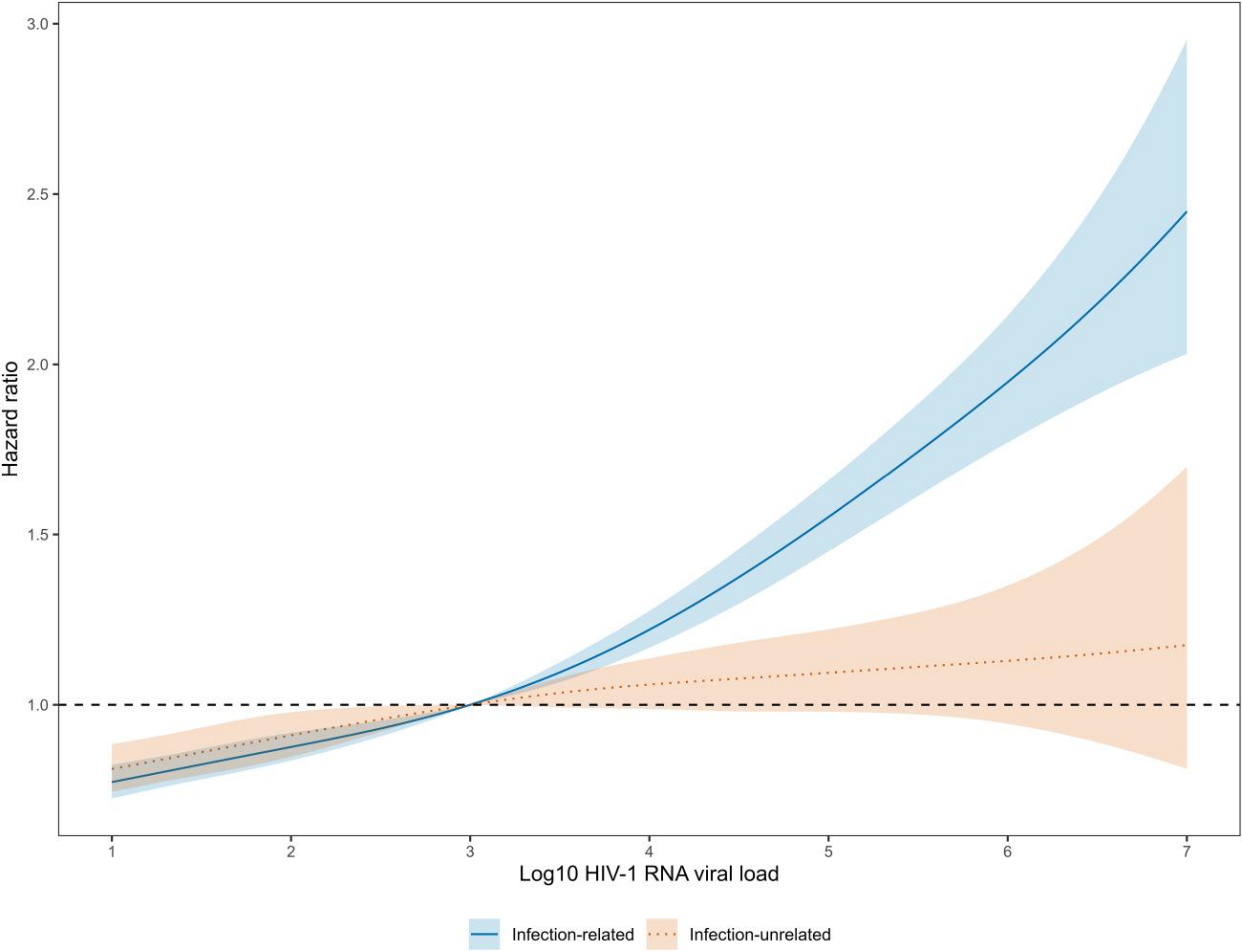
Hazard ratios (lines) with 95% confidence intervals (shaded areas) for the incidence of any infection-related cancer and any infection-unrelated cancer, comparing Log10 HIV-1 RNA viral load values with the reference value of 3. The models were adjusted for sex, age, calendar year, and CD4 cell count (lagged by 6 months). The relationship between Log10 HIV-1 RNA viral load increase and cancer risk was modelled using penalized spline bases with three degrees of freedom. All variables were time-updated. Abbreviation: HIV-1, human immunodeficiency virus type 1.

**Figure 3.**
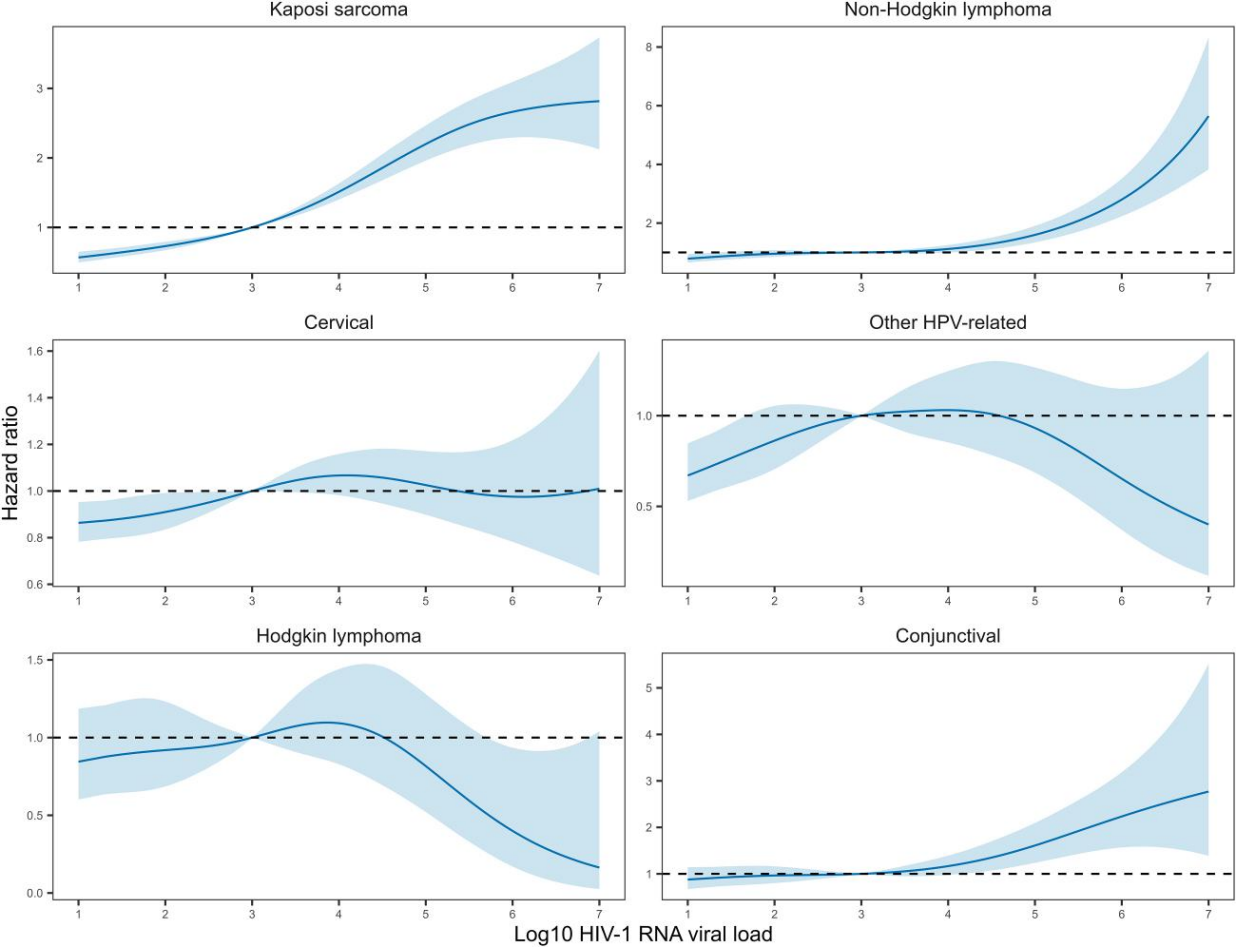
Hazard ratios (solid lines) with 95% confidence intervals (shaded areas) for the incidence of infection-related cancers, comparing a grid of Log10 HIV-1 RNA viral load values with the reference value of 3. The models were adjusted for sex, age, calendar year, and CD4 cell count (lagged by 6 months). The relationship between Log10 HIV-1 RNA viral load increase and cancer risk was modeled using penalized spline bases with 3 degrees of freedom. All variables were time-updated. Abbreviations: HIV-1, human immunodeficiency virus type 1; HPV, human papillomavirus.

**Figure 4.**
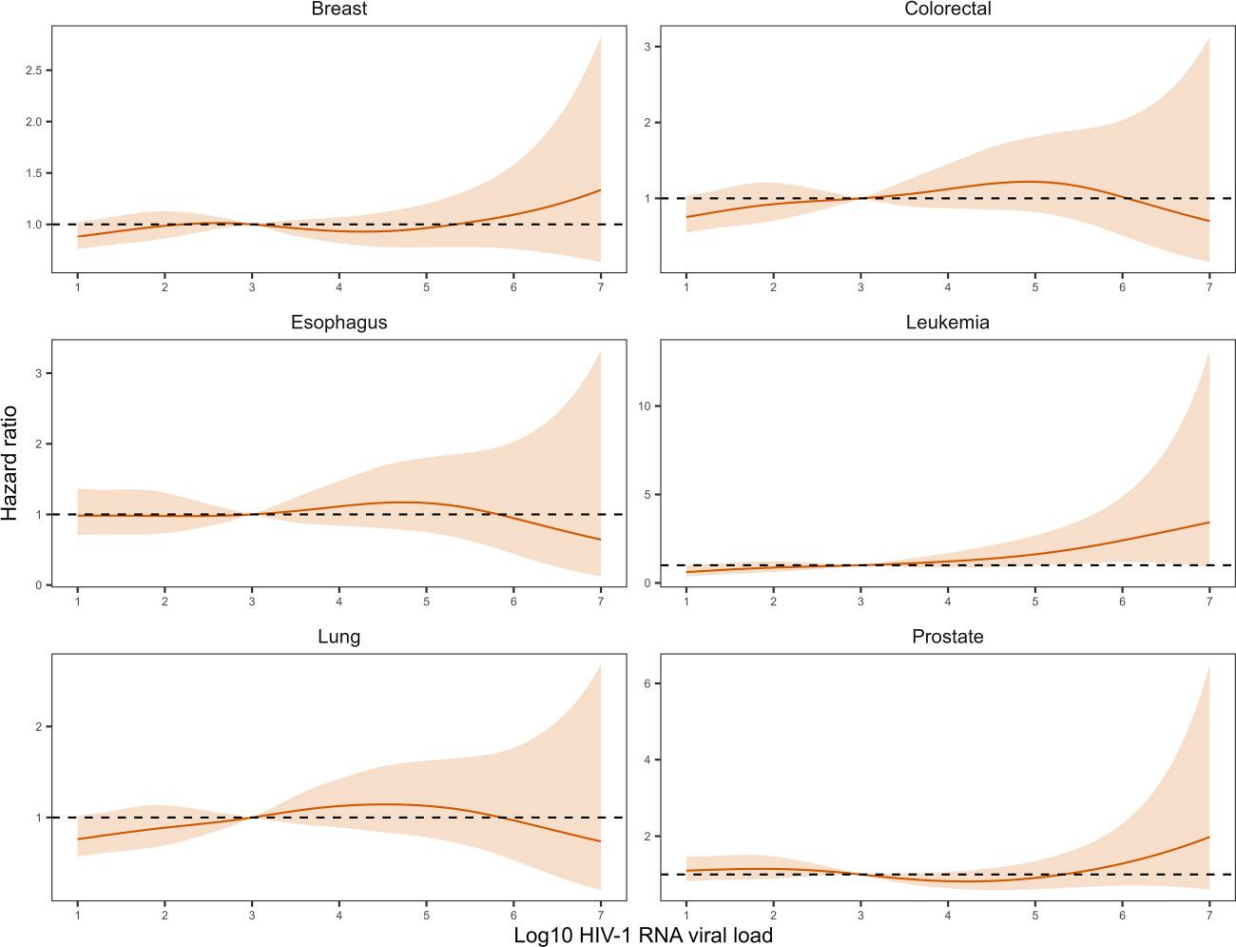
Hazard ratios (solid lines) with 95% confidence intervals (shaded areas) for the incidence of infection-unrelated cancers, comparing Log10 HIV-1 RNA viral load values with the reference value of 3. The models were adjusted for sex, age, calendar year, and CD4 cell count (lagged by 6 months). The relationship between Log10 HIV-1 RNA viral load increase and cancer risk was modelled using penalized spline bases with three degrees of freedom. All variables were time-updated. Abbreviation: HIV-1, human immunodeficiency virus type 1.

In Figures 2–4, we display the adjusted HRs assuming a nonlinear relationship between Log10 HIV-1 RNA viral load increase and cancer risk. Compared with the reference value of 3 Log10 copies/mL, higher viral loads were associated with an increased risk of an infection-related but not infection-unrelated cancer (Figure 2). In contrast, viral loads below the suppression limit were associated with lower incidence rates of both infection-related and infection-unrelated cancers. Increased incidence rates at higher viral load values were apparent for Kaposi sarcoma, non–Hodgkin lymphoma, and conjunctival cancer (Figure 3). The risk of cervical and other HPV-related cancers decreased with declining values below the suppression limit, but there was no clear association with viral loads above the suppression limit. Of note, a very high viral load measurement (>6 Log10 copies/mL) was associated with a lower risk of Hodgkin lymphoma. For most infection-unrelated cancers, no clear association between viral load and incident cancer was visible when assuming a non-linear relationship (Figure 4).

## DISCUSSION

In this cohort study of nearly 2.8 million PWH in South Africa, HIV-1 viremia was associated with an increased risk of infection-related and some infection-unrelated cancers. Kaposi sarcoma, leukemia, non-Hodgkin lymphoma, and conjunctival cancer were most strongly associated with a high HIV-1 RNA viral load. The increased cancer risk remained when accounting for CD4 cell count, suggesting an effect of HIV-1 viremia on the risk of developing these cancers that is not mediated by cellular immunodeficiency.

The large sample size allowed us to examine the association of HIV-1 viremia with less common cancers in PWH. All cancers were pathologically confirmed and coded using International Classification of Diseases for Oncology, 3rd Edition. We assessed cumulative and noncumulative measures of HIV-1 viremia and provided results with and without accounting for immunodeficiency levels. Nevertheless, our study has several limitations. It is based on routine laboratory data and thus only representative for PWH repeatedly accessing care who are likely to have lower HIV-1 RNA levels and a lower cancer risk than those not engaged in care. Furthermore, information on behavioral factors was unavailable and could not be considered. We also lacked information on ART exposure and were unable to determine whether CD4 cell count and HIV-1 RNA viral loads were measured before or after ART initiation. Estimating the independent effect of HIV-1 viremia on cancer risk is challenging given that HIV-1 viremia and cellular immunodeficiency are correlated and HIV-1 viremia is often more precisely measured than immunodeficiency. Differential measurement precision may, thus, have led to an overestimation of the association between HIV-1 viremia and cancer risk in the fully adjusted model [19]. However, when we excluded PWH with severe immunosuppression (<200 CD4 cells/µL) in a sensitivity analysis, the association between HIV-1 viremia and cancer risk became stronger. Furthermore, although we adjusted for CD4 counts to estimate the effect of HIV-1 viremia on cancer risk that was not mediated by immunodeficiency, we did not perform a formal mediation analysis. Adjusting for CD4 count as a potential mediator may also have introduced collider bias if other unmeasured factors are associated with both CD4 count and cancer risk. Nevertheless, conclusions from unadjusted and adjusted models were largely similar. Finally, the median follow-up time in our study was short which limited our ability to quantify a person's cumulative HIV-1 viremia exposure.

Our findings on the positive association of HIV-1 viremia with Kaposi sarcoma and non-Hodgkin lymphoma are largely consistent with previous studies [2, 3, 8, 20, 21]. We also identified HIV-1 viremia as a risk factor for conjunctival cancer, both before and after adjustment for CD4 count. Although conjunctival cancer is classified as an HIV-related malignancy [1], we are not aware of other studies that assessed the relationship between HIV-1 viremia and this cancer. The role of HIV-1 viremia in the development of HPV-related cancers is less clear. Several studies found no association between HIV-1 viremia and the risk of anal cancer [2, 3, 22, 23], whereas Kowalkowski and colleagues reported a greater anal cancer risk with higher cumulative viral loads [10]. Similarly, some studies found an association between HIV-1 viremia and cervical cancer risk [24], whereas others did not [2, 25]. Of note, a systematic review reported HIV-1 viremia to be associated with a higher risk of cervical HPV acquisition and a higher prevalence of anal HPV and precancerous lesions [26]. In our study, we found a weak positive association between HIV-1 viremia and HPV-related cancers. We and others did not find an association between HIV-1 viremia and Hodgkin lymphoma [2, 3, 11]. In contrast, a US-based study reported an increased risk of Hodgkin lymphoma with higher HIV-1 RNA viral load copy-years [10]. Interestingly, HIV-1 viremia was not associated with Hodgkin lymphoma in our study, which may point toward a possible role of HIV-1 viremia in the early rather than late phases of Hodgkin lymphoma development. Most PWH develop Hodgkin lymphoma after ART initiation [27], and it has been hypothesized that the ART-induced repopulation of CD4 cells may provide a favorable microenvironment for the activation of Hodgkin lymphoma–specific Reed-Sternberg cells [28].

In line with others [29, 30], we found HIV-1 viremia to be associated with a higher risk of several infection-unrelated cancers. Compared with HIV-negative US veterans, the relative risk of developing lung cancer, melanoma, and leukemia was higher among unsuppressed than virally suppressed veterans with HIV [11]. The positive association between HIV-1 viremia and infection-unrelated cancers might be partially explained by HIV-induced activation of inflammatory and coagulation pathways [5]. Furthermore, some cancers currently classified as infection-unrelated may indeed have a link with infectious agents, as has been suggested for colorectal cancer and *Fusobacterium nucleatum* [31]. However, differential exposures to traditional cancer risk factors such as smoking, alcohol consumption, and oncogenic viral co-infections between virally suppressed and unsuppressed individuals might confound the observed associations. Of note, some studies found no associations between HIV-1 viremia and the risk of infection-unrelated cancers such as melanoma or lung cancer [2, 32]. In the US veterans study mentioned previously, the prostate cancer risk was lower among unsuppressed than HIV-negative men, wheresa virally suppressed men had an increased risk [11]. We found a slight negative association between HIV-1 viremia and prostate cancer risk in the unadjusted analysis, but this association disappeared in the adjusted models.

HIV-1 viremia may increase cancer risk indirectly through CD4 depletion or more directly through chronic inflammation, accelerated immunosenescence, and direct pro-oncogenic effects of HIV-1 [5–7]. In our analysis, the potential mediating effect of cellular immunodeficiency, inferred from the magnitude of the effect estimate change when adjusting for CD4 count, was largest for Kaposi sarcoma, conjunctival cancer, and non-Hodgkin lymphoma. Similarly, the beneficial effect of ART, which leads to viral suppression and helps restore cellular immunity, has been particularly apparent for Kaposi sarcoma and non-Hodgkin lymphoma [12]. However, the Strategic Timing of Antiretroviral Treatment trial showed that immediate as compared to deferred ART initiation reduced the risk of both infection-related and infection-unrelated cancers [33]. Yet, even among virally suppressed PWH, incidence rates of most cancer types remain higher than among HIV-negative individuals [11]. Furthermore, low-level viremia, typically defined as viral loads between 50 and 999 copies/mL in low- and middle-income settings, is quite common after ART initiation. A South African cohort study found that low-level viremia occurred in almost 1 in 4 PWH on first-line ART [34]. Low-level viremia might lead to adverse clinical outcomes, mediated by residual inflammation and immune activation [35]. Nevertheless, few studies have investigated the impact of low-level viremia on cancer risk, and the available results are inconclusive [8, 13]. In our study, the risk of Kaposi sarcoma, cervical cancer, and other HPV-related cancers decreased further as viral loads dropped <1000 copies/mL, whereas the rates of non-Hodgkin lymphoma, Hodgkin lymphoma, and conjunctival cancer remained stable at viral loads below the suppression limit. Further research is needed to better understand the impact of low-level viremia on cancer rates.

In conclusion, in this cohort of 2.8 million PWH in South Africa, HIV-1 viremia was associated with an increased risk of many infection-related and some infection-unrelated cancers, both before and after controlling for cellular immunodeficiency. For Kaposi sarcoma and HPV-related cancers, the incidence rates further decreased as HIV-1 RNA viral loads dropped from suppressed to undetectable. These observations underline the importance of early initiation of effective ART, regular viral load monitoring, and sustained viral suppression for cancer prevention among PWH in South Africa.

## Supplementary Material

ciae652_Supplementary_Data

## Supplementary Data

Supplementary materials are available at *Clinical Infectious Diseases* online. Consisting of data provided by the authors to benefit the reader, the posted materials are not copyedited and are the sole responsibility of the authors, so questions or comments should be addressed to the corresponding author.

## Notes

***Author Contributions.*** M. M., J. B., E. R., and M. E. conceived the study, recruited staff, and sought funding. Y. R., E. R., and M. E. planned the reported analysis. Y. R. performed the data analysis. Y. R. and E. R. wrote the first draft of the manuscript, which was revised by M. E. All authors reviewed the manuscript and agreed with the final version. All authors had full access to all the data in the study and had final responsibility for the decision to submit for publication.

***Financial support.*** This work was supported by the National Institute of Allergy and Infectious Diseases of the U.S. National Institutes of Health (NIH) under Award Number U01AI069924. The work was also supported by a PEPFAR supplement (to M. E.), the Swiss National Science Foundation (SNSF, 320030_169967 to J. B., M. E.), and the U.S. CRDF Global (HIV_DAA3-16-62705-1 to M. M.). M. E. was supported by special project funding (grant 32FP30-189498) from the SNSF. Calculations were performed on UBELIX (http://www.id.unibe.ch/hpc), the HPC cluster at the University of Bern.

***Data sharing.*** The data underlying this article will be shared on reasonable request to the corresponding author, subject to approval by the SAM cohort scientific committee.
