# Supplementary material for: HIV-1 Viremia and Cancer Risk in 2.8 Million People: The South African HIV Cancer Match Study
Source: Clin Infect Dis. 2024 Dec 30;81(2):286–95. doi: 10.1093/cid/ciae652 (PMC12448642; doi:10.1093/cid/ciae652)
Supplement: ciae652_Supplementary_Data [file ciae652_supplementary_data.docx]

**HIV-1 viremia and Cancer Risk in 2.8 Million People:**

**the South African HIV Cancer Match Study**

**Supplementary material**

**Supplemental Table 1**: Cancer classification based on the International Classification of Diseases for Oncology, 3^rd^ Edition (ICD-0-3).

**Supplemental Table 2:** The different measures and mathematical transformations of HIV-1 viremia considered for analysis. All variables were time-updated.

**Supplemental Table 3:** Unadjusted hazard ratios and 95% confidence intervals quantifying the association between HIV-1 viremia and the incidence of specific infection-related cancers and any infection-related cancer, using different measures of HIV-1 viremia. All variables were time-updated.

**Supplemental Table 4:** Unadjusted hazard ratios and 95% confidence intervals quantifying the association between HIV-1 viremia and the incidence of specific infection-unrelated cancers and any infection-unrelated cancer, using different measures of HIV-1 viremia. All variables were time-updated.

**Supplemental Table 5:** Hazard ratios and 95% confidence intervals quantifying the association between HIV-1 viremia and the incidence of specific infection-related cancers and any infection-related cancer, using different measures of HIV-1 viremia. Models were adjusted for age, sex, and calendar year. All variables were time-updated.

**Supplemental Table 6:** Hazard ratios and 95% confidence intervals quantifying the association between HIV-1 viremia and the incidence of specific infection-unrelated cancers and any infection-unrelated cancer, using different measures of HIV-1 viremia. Models were adjusted for age, sex, and calendar year. All variables were time-updated.

**Supplemental Table 7:** Hazard ratios and 95% confidence intervals quantifying the association between HIV-1 viremia and the incidence of specific infection-related cancers and any infection-related cancer, using different measures of HIV-1 viremia. Models were adjusted for age, sex, calendar year, and CD4 cell count (lagged by six months). All variables were time-updated.

**Supplemental Table 8:** Hazard ratios and 95% confidence intervals quantifying the association between HIV-1 viremia and the incidence of specific infection-unrelated cancers and any infection-unrelated cancer, using different measures of HIV-1 viremia. Models were adjusted for age, sex, calendar year, and CD4 cell count (lagged by six months). All variables were time-updated.

**Supplemental Table 9:** Hazard ratios and 95% confidence intervals quantifying the association between HIV-1 viremia and the incidence of specific infection-related cancers and any infection-related cancer, using different measures of HIV-1 viremia. Models were adjusted for age, sex, calendar year, and CD4 cell count (lagged by six months). All variables were time-updated. Individuals with no CD4 cell count measurement at or before the baseline visit were included in the study population.

**Supplemental Table 10:** Hazard ratios and 95% confidence intervals quantifying the association between HIV-1 viremia and the incidence of specific infection-unrelated cancers and any infection-unrelated cancer, using different measures of HIV-1 viremia. Models were adjusted for age, sex, calendar year, and CD4 cell count (lagged by six months). All variables were time-updated. Individuals with no CD4 cell count measurement at or before the baseline visit were included in the study population.

**Supplemental Table 11:** Hazard ratios and 95% confidence intervals quantifying the association between HIV-1 viremia and the incidence of specific infection-related cancers and any infection-related cancer, using different measures of HIV-1 viremia. Models were adjusted for age, sex, calendar year, and CD4 cell count (lagged by six months). All variables were time-updated. Time-at-risk with no available 6-month lagged CD4 cell count was removed from the analysis.

**Supplemental Table 12:** Hazard ratios and 95% confidence intervals quantifying the association between HIV-1 viremia and the incidence of specific infection-unrelated cancers and any infection-unrelated cancer, using different measures of HIV-1 viremia. Models were adjusted for age, sex, calendar year, and CD4 cell count (lagged by six months). All variables were time-updated. Time-at-risk with no available 6-month lagged CD4 cell count was removed from the analysis.

**Supplemental Table 13:** Hazard ratios and 95% confidence intervals quantifying the association between HIV-1 viremia and the incidence of specific infection-related cancers and any infection-related cancer, using different measures of HIV-1 viremia. Models were adjusted for age, sex, and calendar year. Individuals with CD4 cell counts <200 cells/µl at any time point were excluded from this analysis.

**Supplemental Table 14:** Hazard ratios and 95% confidence intervals quantifying the association between HIV-1 viremia and the incidence of specific infection-unrelated cancers and any infection-unrelated cancer, using different measures of HIV-1 viremia. Models were adjusted for age, sex, and calendar year. Individuals with CD4 cell counts <200 cells/µl at any time point were excluded from this analysis.

**Supplemental Figure 1:** Flowchart showing the selection of the study population.

**Supplemental Table 1**: Cancer classification based on the International Classification of Diseases for Oncology, 3^rd^ Edition (ICD-0-3).

| **Category/cancer** | **ICD-0-3 codes** |
| --- | --- |
| **Infection-related cancers** | |
| Cervical | Topographies C53.0-C53.9* |
| Kaposi sarcoma | Morphology 9140 |
| Non-Hodgkin’s lymphoma | Morphologies 9590, 9591, 9596, 9670, 9671, 9673 |
| HPV-related, excluding cervical cancer |  |
| Anal | Topographies C21.0-C21.9* |
| Head and neck | Topographies C01.9, C02.4, C09.0-C09.9, C10.2-C10.9, C14.0, C14.2* |
| Penile | Topographies C60.0-C60.9* |
| Vaginal | Topography C52.9* |
| Vulvar | Topographies C51.0-51.9* |
| Conjunctival | Topography C69.0* |
| Hodgkin lymphoma | Morphologies 9650-9654, 9655, 9659, 9663, 9664, 9665, 9667 |
| Nasopharyngeal | Topographies C11.0-C11.9 |
| Stomach | Topographies C16.0-C16.9* |
| Bladder | Topographies C69.9, C67.0-C67.9* |
| Liver and bile duct | Topographies C22.0 C22.1, C23.9, C24.1, C24.9* |
| **Infection-unrelated cancers** | |
| Breast | Topographies C50.0-C50.9* |
| Colorectal | Topographies C18.0-C18.9, C19.9, C20.9* |
| Esophagus | Topographies C15.0-15.9* |
| Leukemia | Morphologies 9800, 9801, 9805, 9820, 9823, 9826, 9827, 9832, 9833-9837, 9840, 9860, 9861, 9863, 9866, 9867, 9870-9873, 9874-9876, 9891, 9895, 9896, 9910, 9930, 9931, 9940, 9945, 9946 |
| Lung | Topographies C33.9, C34.0-C34.9* |
| Prostate | Topography C61.9* |
| Other infection-unrelated | Various** |

HPV: human papillomavirus; NHL: non-Hodgkin lymphoma * excluding morphologies indicating Kaposi sarcomas, non-Hodgkin lymphoma, and Hodgkin lymphoma; ** includes all cancers not mentioned in the table, except ill-defined cancers (topography codes C76.0-C76.7) and cancers with unknown primary site (topography code C80.9)

**Supplemental Table 2:** The different measures and mathematical transformations of HIV-1 viremia considered for analysis. All variables were time-updated.

| **Measure** | **Variable type** | **Categories or transformation** | **Measure of relative cancer risk** |
| --- | --- | --- | --- |
| HIV-1 RNA viral load | Continuous | Log10 transformation | HR per unit increase in Log10 copies/mL (equivalently, per 10-fold increase in copies/mL). |
|  |  | Log10 and penalized spline transformation with 3 degrees of freedom | HR over grid of values, compared with reference value of 3 Log10 copies/mL |
| Percentage time virally suppressed (<1,000 HIV-1 RNA viral load copies/mL) | Categorical | 0-19%, 20-49%, 50-79%, 80-100% | HR compared with reference category of 80-100% |
| Cumulative HIV-1 RNA viral load | Categorical | 0-9, 10-99, 100-999, 1,000-9,999, 10,000-99,999, ≥100,000 copy-years/mL | HR compared with reference category of 0-9 copy-years/mL |
|  | Ordinal* |  | HR per Log10 unit increase in copy-years/mL (equivalently, per 10-fold increase in copy-years/mL). * |

HR: hazard ratio; * treating the categorical variable as a continuous variable, 1 corresponds to category 0-9, 2 to 10-99, 3 to 100-999, etc.

**Supplemental Table 3:** Unadjusted hazard ratios and 95% confidence intervals quantifying the association between HIV-1 viremia and the incidence of specific infection-related cancers and any infection-related cancer, using different measures of HIV-1 viremia. All variables were time-updated.

|  | **Kaposi Sarcoma** | **Non-Hodgkin lymphoma** | **Cervical cancer** | **Other HPV-related**  **cancers** | **Hodgkin lymphoma** | **Conjunctival  cancer** | **Any infection-related cancer** |
| --- | --- | --- | --- | --- | --- | --- | --- |
| **Per Log10 HIV-1 RNA viral load unit increase (copy/mL)** | 1.56 (1.52-1.60) | 1.31 (1.26-1.36) | 1.04 (1.01-1.07) | 1.09 (1.03-1.17) | 1.04 (0.95-1.14) | 1.34 (1.26-1.42) | 1.25 (1.23-1.26) |
| **Percentage time virally suppressed** |  |  |  |  |  |  |  |
| 80-100% (ref) | 1 | 1 | 1 | 1 | 1 | 1 | 1 |
| 50-79% | 2.08 (1.65-2.64) | 1.94 (1.48-2.53) | 1.31 (1.14-1.50) | 0.96 (0.68-1.35) | 0.75 (0.41-1.38) | 1.90 (1.31-2.76) | 1.50 (1.36-1.65) |
| 20-49% | 3.23 (2.56-4.07) | 1.63 (1.15-2.31) | 1.26 (1.06-1.49) | 1.53 (1.07-2.19) | 0.97 (0.49-1.93) | 2.62 (1.76-3.88) | 1.67 (1.50-1.87) |
| 0-19% | 4.75 (4.30-5.25) | 2.17 (1.88-2.51) | 1.30 (1.17-1.43) | 1.37 (1.09-1.74) | 1.30 (0.94-1.79) | 2.94 (2.37-3.66) | 2.20 (2.09-2.32) |
| **Cumulative HIV-1 RNA viral load (copy-years/mL)** |  |  |  |  |  |  |  |
| 0-9 (ref) | 1 | 1 | 1 | 1 | 1 | 1 | 1 |
| 10-99 | 1.62 (1.34-1.96) | 1.41 (1.10-1.80) | 1.04 (0.89-1.21) | 1.48 (0.98-2.23) | 1.46 (0.91-2.36) | 0.95 (0.64-1.41) | 1.42 (1.30-1.57) |
| 100-999 | 2.39 (1.99-2.86) | 1.68 (1.31-2.15) | 0.98 (0.84-1.14) | 1.60 (1.07-2.39) | 1.29 (0.78-2.14) | 1.06 (0.71-1.57) | 1.62 (1.48-1.78) |
| 1,000-9,999 | 4.72 (4.01-5.55) | 2.22 (1.74-2.84) | 1.10 (0.94-1.30) | 1.40 (0.91-2.15) | 1.82 (1.11-2.99) | 1.41 (0.94-2.13) | 2.20 (2.00-2.42) |
| 10,000-99,999 | 7.33 (6.23-8.62) | 3.25 (2.56-4.13) | 1.19 (1.01-1.40) | 1.46 (0.95-2.25) | 1.08 (0.62-1.89) | 2.31 (1.60-3.34) | 2.70 (2.46-2.96) |
| ≥ 100,000 | 11.82 (9.87-14.16) | 4.17 (3.18-5.47) | 1.24 (1.04-1.48) | 1.92 (1.23-2.99) | 1.15 (0.61-2.19) | 4.77 (3.28-6.93) | 3.29 (2.97-3.65) |
| **Per Log10 cumulative HIV-1 RNA viral load unit increase (copy-years/mL)** | 1.66 (1.61-1.71) | 1.33 (1.27-1.39) | 1.05 (1.02-1.08) | 1.07 (1.00-1.14) | 1.01 (0.91-1.11) | 1.41 (1.32-1.50) | 1.26 (1.24-1.28) |

HPV: human papillomavirus

**Supplemental Table 4:** Unadjusted hazard ratios and 95% confidence intervals quantifying the association between HIV-1 viremia and the incidence of specific infection-unrelated cancers and any infection-unrelated cancer, using different measures of HIV-1 viremia. All variables were time-updated.

|  | **Breast cancer** | **Colorectal cancer** | **Esophageal cancer** | **Leukemia** | **Lung cancer** | **Prostate cancer** | **Any infection-unrelated cancer** |
| --- | --- | --- | --- | --- | --- | --- | --- |
| **Per Log10 HIV-1 RNA viral load unit increase (copy/mL)** | 0.98 (0.94-1.03) | 1.07 (0.98-1.16) | 0.99 (0.90-1.08) | 1.26 (1.12-1.41) | 1.07 (0.99-1.16) | 0.91 (0.84-0.99) | 1.05 (1.03-1.07) |
| **Percentage time virally suppressed** |  |  |  |  |  |  |  |
| 80-100% (ref) | 1 | 1 | 1 | 1 | 1 | 1 | 1 |
| 50-79% | 0.96 (0.78-1.19) | 0.75 (0.44-1.26) | 1.28 (0.84-1.96) | 0.54 (0.20-1.52) | 1.17 (0.78-1.76) | 0.87 (0.59-1.29) | 1.10 (0.98-1.25) |
| 20-49% | 0.87 (0.66-1.15) | 0.87 (0.47-1.61) | 1.01 (0.56-1.83) | 0.74 (0.23-2.39) | 1.01 (0.58-1.75) | 0.86 (0.52-1.42) | 1.05 (0.90-1.22) |
| 0-19% | 0.87 (0.73-1.04) | 1.36 (1.01-1.84) | 0.87 (0.61-1.25) | 1.88 (1.26-2.82) | 1.34 (1.01-1.78) | 0.65 (0.46-0.91) | 1.19 (1.09-1.29) |
| **Cumulative HIV-1 RNA viral load (copy-years/mL)** |  |  |  |  |  |  |  |
| 0-9 (ref) | 1 | 1 | 1 | 1 | 1 | 1 | 1 |
| 10-99 | 0.99 (0.77-1.27) | 1.18 (0.72-1.94) | 0.86 (0.52-1.43) | 1.70 (0.86-3.33) | 1.12 (0.71-1.74) | 0.76 (0.48-1.21) | 1.07 (0.93-1.23) |
| 100-999 | 0.98 (0.76-1.27) | 1.46 (0.90-2.36) | 1.01 (0.62-1.66) | 2.19 (1.13-4.24) | 1.02 (0.65-1.61) | 0.79 (0.51-1.24) | 1.19 (1.04-1.37) |
| 1,000-9,999 | 0.94 (0.71-1.23) | 1.03 (0.59-1.78) | 0.78 (0.45-1.36) | 2.00 (0.98-4.08) | 1.05 (0.65-1.70) | 0.77 (0.47-1.24) | 1.21 (1.05-1.40) |
| 10,000-99,999 | 0.82 (0.63-1.08) | 1.51 (0.90-2.51) | 0.65 (0.37-1.15) | 2.06 (0.97-4.38) | 1.30 (0.82-2.07) | 0.50 (0.30-0.83) | 1.13 (0.98-1.31) |
| ≥ 100,000 | 0.87 (0.64-1.17) | 1.28 (0.71-2.29) | 0.95 (0.53-1.71) | 3.29 (1.47-7.34) | 1.25 (0.75-2.10) | 0.59 (0.35-0.98) | 1.19 (1.02-1.40) |
| **Per Log10 cumulative HIV-1 RNA viral load unit increase (copy-years/mL)** | 0.96 (0.92-1.00) | 1.04 (0.95-1.14) | 0.96 (0.87-1.05) | 1.18 (1.04-1.35) | 1.05 (0.97-1.14) | 0.90 (0.83-0.97) | 1.03 (1.00-1.05) |

**Supplemental Table 5:** Hazard ratios and 95% confidence intervals quantifying the association between HIV-1 viremia and the incidence of specific infection-related cancers and any infection-related cancer, using different measures of HIV-1 viremia. Models were adjusted for age, sex, and calendar year. All variables were time-updated.

|  | **Kaposi Sarcoma** | **Non-Hodgkin lymphoma** | **Cervical cancer** | **Other HPV-related**  **cancers** | **Hodgkin lymphoma** | **Conjunctival  cancer** | **Any infection-related cancer** |
| --- | --- | --- | --- | --- | --- | --- | --- |
| **Per Log10 HIV-1 RNA viral load unit increase (copy/mL)** | 1.46 (1.42-1.50) | 1.27 (1.22-1.33) | 1.07 (1.04-1.10) | 1.11 (1.04-1.19) | 0.99 (0.91-1.09) | 1.32 (1.25-1.41) | 1.24 (1.22-1.25) |
| **Percentage time virally suppressed** |  |  |  |  |  |  |  |
| 80-100% (ref) | 1 | 1 | 1 | 1 | 1 | 1 | 1 |
| 50-79% | 1.67 (1.32-2.11) | 1.75 (1.33-2.29) | 1.34 (1.17-1.53) | 0.99 (0.70-1.39) | 0.66 (0.36-1.21) | 1.69 (1.16-2.46) | 1.40 (1.27-1.54) |
| 20-49% | 2.60 (2.06-3.28) | 1.49 (1.05-2.12) | 1.34 (1.13-1.60) | 1.61 (1.13-2.30) | 0.85 (0.43-1.69) | 2.40 (1.62-3.58) | 1.60 (1.43-1.79) |
| 0-19% | 3.79 (3.42-4.20) | 1.98 (1.70-2.30) | 1.44 (1.30-1.60) | 1.48 (1.16-1.88) | 1.09 (0.78-1.52) | 2.81 (2.24-3.51) | 2.13 (2.01-2.25) |
| **Cumulative HIV-1 RNA viral load (copy-years/mL)** |  |  |  |  |  |  |  |
| 0-9 (ref) | 1 | 1 | 1 | 1 | 1 | 1 | 1 |
| 10-99 | 1.50 (1.24-1.81) | 1.34 (1.05-1.72) | 1.03 (0.89-1.21) | 1.50 (0.99-2.26) | 1.37 (0.85-2.21) | 0.93 (0.62-1.39) | 1.39 (1.26-1.52) |
| 100-999 | 2.09 (1.74-2.50) | 1.58 (1.24-2.03) | 1.00 (0.86-1.17) | 1.64 (1.09-2.46) | 1.17 (0.71-1.93) | 1.04 (0.70-1.55) | 1.58 (1.44-1.73) |
| 1,000-9,999 | 3.65 (3.09-4.30) | 2.00 (1.56-2.57) | 1.19 (1.01-1.40) | 1.48 (0.96-2.29) | 1.50 (0.91-2.47) | 1.33 (0.88-2.01) | 2.07 (1.88-2.28) |
| 10,000-99,999 | 5.24 (4.44-6.19) | 2.78 (2.18-3.54) | 1.29 (1.10-1.52) | 1.58 (1.02-2.44) | 0.82 (0.46-1.45) | 2.12 (1.46-3.09) | 2.49 (2.26-2.74) |
| ≥ 100,000 | 8.01 (6.67-9.63) | 3.45 (2.62-4.55) | 1.36 (1.13-1.63) | 2.09 (1.33-3.28) | 0.83 (0.43-1.58) | 4.36 (2.98-6.39) | 3.07 (2.76-3.41) |
| **Per Log10 cumulative HIV-1 RNA viral load unit increase (copy-years/mL)** | 1.53 (1.48-1.57) | 1.28 (1.22-1.34) | 1.07 (1.04-1.11) | 1.09 (1.02-1.17) | 0.94 (0.85-1.04) | 1.38 (1.29-1.48) | 1.24 (1.22-1.26) |

HPV: human papillomavirus

**Supplemental Table 6:** Hazard ratios and 95% confidence intervals quantifying the association between HIV-1 viremia and the incidence of specific infection-unrelated cancers and any infection-unrelated cancer, using different measures of HIV-1 viremia. Models were adjusted for age, sex, and calendar year. All variables were time-updated.

|  | **Breast cancer** | **Colorectal cancer** | **Esophageal cancer** | **Leukemia** | **Lung cancer** | **Prostate cancer** | **Any infection-unrelated cancer** |
| --- | --- | --- | --- | --- | --- | --- | --- |
| **Per Log10 HIV-1 RNA viral load unit increase (copy/mL)** | 1.03 (0.99-1.08) | 1.10 (1.01-1.20) | 1.02 (0.93-1.12) | 1.21 (1.07-1.35) | 1.08 (1.00-1.17) | 0.96 (0.88-1.05) | 1.08 (1.06-1.11) |
| **Percentage time virally suppressed** |  |  |  |  |  |  |  |
| 80-100% (ref) | 1 | 1 | 1 | 1 | 1 | 1 | 1 |
| 50-79% | 1.02 (0.82-1.26) | 0.79 (0.47-1.34) | 1.37 (0.89-2.09) | 0.44 (0.16-1.23) | 1.22 (0.82-1.84) | 0.97 (0.66-1.44) | 1.14 (1.01-1.28) |
| 20-49% | 0.98 (0.74-1.30) | 0.96 (0.52-1.79) | 1.14 (0.63-2.06) | 0.63 (0.19-2.01) | 1.10 (0.63-1.91) | 1.02 (0.62-1.68) | 1.14 (0.97-1.33) |
| 0-19% | 1.05 (0.88-1.25) | 1.56 (1.15-2.11) | 1.02 (0.71-1.47) | 1.56 (1.03-2.37) | 1.45 (1.09-1.94) | 0.79 (0.56-1.12) | 1.32 (1.21-1.45) |
| **Cumulative HIV-1 RNA viral load (copy-years/mL)** |  |  |  |  |  |  |  |
| 0-9 (ref) | 1 | 1 | 1 | 1 | 1 | 1 | 1 |
| 10-99 | 0.99 (0.77-1.28) | 1.17 (0.71-1.92) | 0.84 (0.51-1.39) | 1.54 (0.78-3.02) | 1.05 (0.67-1.64) | 0.76 (0.48-1.20) | 1.05 (0.91-1.21) |
| 100-999 | 1.03 (0.80-1.33) | 1.48 (0.91-2.40) | 1.01 (0.62-1.67) | 1.90 (0.98-3.69) | 0.97 (0.61-1.53) | 0.80 (0.51-1.26) | 1.20 (1.04-1.38) |
| 1,000-9,999 | 1.08 (0.82-1.42) | 1.15 (0.66-2.01) | 0.88 (0.51-1.54) | 1.57 (0.76-3.22) | 1.09 (0.67-1.78) | 0.86 (0.53-1.41) | 1.31 (1.13-1.51) |
| 10,000-99,999 | 0.98 (0.74-1.29) | 1.70 (1.01-2.85) | 0.74 (0.42-1.31) | 1.47 (0.68-3.18) | 1.32 (0.83-2.11) | 0.62 (0.37-1.02) | 1.23 (1.06-1.42) |
| ≥ 100,000 | 1.05 (0.77-1.42) | 1.41 (0.78-2.55) | 1.05 (0.59-1.89) | 2.27 (1.00-5.14) | 1.20 (0.72-2.02) | 0.80 (0.48-1.34) | 1.30 (1.10-1.52) |
| **Per Log10 cumulative HIV-1 RNA viral load unit increase (copy-years/mL)** | 1.01 (0.96-1.05) | 1.08 (0.98-1.18) | 0.99 (0.90-1.09) | 1.10 (0.96-1.25) | 1.06 (0.97-1.15) | 0.96 (0.88-1.04) | 1.05 (1.03-1.08) |

HPV: human papillomavirus

**Supplemental Table 7:** Hazard ratios and 95% confidence intervals quantifying the association between HIV-1 viremia and the incidence of specific infection-related cancers and any infection-related cancer, using different measures of HIV-1 viremia. Models were adjusted for age, sex, calendar year, and CD4 cell count (lagged by six months). All variables were time-updated.

|  | **Kaposi Sarcoma** | **Non-Hodgkin lymphoma** | **Cervical cancer** | **Other HPV-related**  **cancers** | **Hodgkin lymphoma** | **Conjunctival  cancer** | **Any infection-related cancer** |
| --- | --- | --- | --- | --- | --- | --- | --- |
| **Per Log10 HIV-1 RNA viral load unit increase (copy/mL)** | 1.38 (1.35-1.42) | 1.24 (1.19-1.29) | 1.05 (1.02-1.08) | 1.09 (1.02-1.16) | 0.99 (0.90-1.08) | 1.18 (1.11-1.25) | 1.19 (1.17-1.21) |
| **Percentage time virally suppressed** |  |  |  |  |  |  |  |
| 80-100% (ref) | 1 | 1 | 1 | 1 | 1 | 1 | 1 |
| 50-79% | 1.60 (1.26-2.03) | 1.68 (1.28-2.20) | 1.30 (1.13-1.49) | 0.93 (0.66-1.32) | 0.64 (0.35-1.18) | 1.48 (1.02-2.16) | 1.35 (1.23-1.49) |
| 20-49% | 2.21 (1.75-2.79) | 1.34 (0.94-1.90) | 1.25 (1.05-1.49) | 1.46 (1.01-2.10) | 0.82 (0.41-1.64) | 1.66 (1.11-2.49) | 1.44 (1.28-1.61) |
| 0-19% | 3.20 (2.88-3.55) | 1.78 (1.52-2.08) | 1.33 (1.20-1.48) | 1.35 (1.05-1.73) | 1.07 (0.76-1.50) | 1.84 (1.46-2.32) | 1.88 (1.78-2.00) |
| **Cumulative HIV-1 RNA viral load (copy-years/mL)** |  |  |  |  |  |  |  |
| 0-9 (ref) | 1 | 1 | 1 | 1 | 1 | 1 | 1 |
| 10-99 | 1.46 (1.21-1.76) | 1.33 (1.04-1.70) | 1.02 (0.88-1.19) | 1.51 (1.00-2.27) | 1.37 (0.85-2.21) | 0.91 (0.61-1.35) | 1.36 (1.24-1.49) |
| 100-999 | 2.01 (1.68-2.41) | 1.55 (1.21-1.99) | 0.98 (0.84-1.15) | 1.64 (1.09-2.46) | 1.16 (0.70-1.92) | 1.01 (0.68-1.50) | 1.53 (1.39-1.68) |
| 1,000-9,999 | 3.26 (2.76-3.85) | 1.89 (1.47-2.42) | 1.13 (0.96-1.34) | 1.44 (0.93-2.23) | 1.46 (0.88-2.41) | 1.11 (0.73-1.68) | 1.92 (1.75-2.11) |
| 10,000-99,999 | 4.32 (3.65-5.11) | 2.49 (1.95-3.20) | 1.19 (1.00-1.40) | 1.45 (0.93-2.26) | 0.78 (0.44-1.38) | 1.47 (1.00-2.15) | 2.18 (1.98-2.40) |
| ≥ 100,000 | 6.03 (4.99-7.30) | 2.94 (2.21-3.90) | 1.19 (0.99-1.44) | 1.83 (1.15-2.91) | 0.76 (0.39-1.48) | 2.47 (1.66-3.68) | 2.50 (2.25-2.79) |
| **Per Log10 cumulative HIV-1 RNA viral load unit increase (copy-years/mL)** | 1.44 (1.39-1.49) | 1.24 (1.18-1.30) | 1.05 (1.01-1.08) | 1.06 (0.98-1.14) | 0.93 (0.84-1.03) | 1.21 (1.13-1.30) | 1.19 (1.17-1.21) |

HPV: human papillomavirus

**Supplemental Table 8:** Hazard ratios and 95% confidence intervals quantifying the association between HIV-1 viremia and the incidence of specific infection-unrelated cancers and any infection-unrelated cancer, using different measures of HIV-1 viremia. Models were adjusted for age, sex, calendar year, and CD4 cell count (lagged by six months). All variables were time-updated.

|  | **Breast cancer** | **Colorectal cancer** | **Esophageal cancer** | **Leukemia** | **Lung cancer** | **Prostate cancer** | **Any infection-unrelated cancer** |
| --- | --- | --- | --- | --- | --- | --- | --- |
| **Per Log10 HIV-1 RNA viral load unit increase (copy/mL)** | 1.04 (0.99-1.09) | 1.11 (1.02-1.21) | 1.02 (0.93-1.13) | 1.28 (1.13-1.45) | 1.09 (1.01-1.19) | 0.97 (0.89-1.06) | 1.08 (1.05-1.11) |
| **Percentage time virally suppressed** |  |  |  |  |  |  |  |
| 80-100% (ref) | 1 | 1 | 1 | 1 | 1 | 1 | 1 |
| 50-79% | 1.01 (0.82-1.25) | 0.80 (0.47-1.35) | 1.37 (0.89-2.10) | 0.49 (0.18-1.38) | 1.24 (0.83-1.87) | 0.99 (0.67-1.47) | 1.14 (1.01-1.29) |
| 20-49% | 0.98 (0.74-1.31) | 0.98 (0.53-1.84) | 1.14 (0.62-2.07) | 0.77 (0.24-2.50) | 1.14 (0.65-1.98) | 1.06 (0.64-1.76) | 1.14 (0.97-1.33) |
| 0-19% | 1.05 (0.88-1.27) | 1.62 (1.18-2.23) | 1.02 (0.70-1.49) | 1.90 (1.22-2.96) | 1.51 (1.12-2.05) | 0.83 (0.58-1.19) | 1.32 (1.20-1.45) |
| **Cumulative HIV-1 RNA viral load (copy-years/mL)** |  |  |  |  |  |  |  |
| 0-9 (ref) | 1 | 1 | 1 | 1 | 1 | 1 | 1 |
| 10-99 | 1.00 (0.77-1.28) | 1.18 (0.72-1.95) | 0.84 (0.51-1.39) | 1.56 (0.79-3.07) | 1.05 (0.67-1.65) | 0.76 (0.48-1.21) | 1.04 (0.91-1.20) |
| 100-999 | 1.03 (0.80-1.33) | 1.50 (0.92-2.45) | 1.01 (0.61-1.67) | 1.98 (1.01-3.85) | 0.97 (0.61-1.54) | 0.81 (0.52-1.27) | 1.19 (1.04-1.37) |
| 1,000-9,999 | 1.08 (0.82-1.42) | 1.18 (0.67-2.05) | 0.88 (0.50-1.54) | 1.77 (0.85-3.68) | 1.11 (0.68-1.81) | 0.88 (0.54-1.44) | 1.30 (1.12-1.50) |
| 10,000-99,999 | 0.98 (0.74-1.30) | 1.76 (1.04-2.97) | 0.73 (0.41-1.31) | 1.83 (0.84-4.02) | 1.36 (0.84-2.18) | 0.64 (0.38-1.07) | 1.21 (1.04-1.40) |
| ≥ 100,000 | 1.05 (0.77-1.43) | 1.49 (0.81-2.73) | 1.05 (0.57-1.91) | 3.09 (1.33-7.18) | 1.25 (0.74-2.14) | 0.85 (0.51-1.45) | 1.27 (1.07-1.49) |
| **Per Log10 cumulative HIV-1 RNA viral load unit increase (copy-years/mL)** | 1.01 (0.96-1.06) | 1.09 (0.99-1.20) | 0.99 (0.90-1.09) | 1.17 (1.02-1.35) | 1.07 (0.98-1.16) | 0.97 (0.89-1.06) | 1.05 (1.02-1.08) |

**Supplemental Table 9:** Hazard ratios and 95% confidence intervals quantifying the association between HIV-1 viremia and the incidence of specific infection-related cancers and any infection-related cancer, using different measures of HIV-1 viremia. Models were adjusted for age, sex, calendar year, and CD4 cell count (lagged by six months). All variables were time-updated. Individuals with no CD4 cell count measurement at or before the baseline visit were included in the study population.

|  | **Kaposi Sarcoma** | **Non-Hodgkin lymphoma** | **Cervical cancer** | **Other HPV-related**  **cancers** | **Hodgkin lymphoma** | **Conjunctival  cancer** | **Any infection-related cancer** |
| --- | --- | --- | --- | --- | --- | --- | --- |
| **Per Log10 HIV-1 RNA viral load unit increase (copy/mL)** | 1.37 (1.33-1.40) | 1.21 (1.17-1.26) | 1.04 (1.01-1.07) | 1.07 (1.00-1.14) | 0.98 (0.90-1.08) | 1.17 (1.11-1.24) | 1.18 (1.16-1.20) |
| **Percentage time virally suppressed** |  |  |  |  |  |  |  |
| 80-100% (ref) | 1 | 1 | 1 | 1 | 1 | 1 | 1 |
| 50-79% | 1.55 (1.24-1.93) | 1.52 (1.18-1.97) | 1.27 (1.11-1.44) | 1.03 (0.76-1.40) | 0.55 (0.31-0.99) | 1.52 (1.08-2.15) | 1.31 (1.20-1.43) |
| 20-49% | 2.15 (1.72-2.67) | 1.44 (1.05-1.97) | 1.25 (1.06-1.47) | 1.40 (1.00-1.97) | 0.97 (0.54-1.73) | 1.62 (1.11-2.37) | 1.44 (1.29-1.59) |
| 0-19% | 3.17 (2.87-3.51) | 1.71 (1.47-1.99) | 1.30 (1.18-1.44) | 1.32 (1.04-1.68) | 1.02 (0.73-1.42) | 1.85 (1.48-2.30) | 1.85 (1.75-1.95) |
| **Cumulative HIV-1 RNA viral load (copy-years/mL)** |  |  |  |  |  |  |  |
| 0-9 (ref) | 1 | 1 | 1 | 1 | 1 | 1 | 1 |
| 10-99 | 1.57 (1.31-1.88) | 1.32 (1.04-1.68) | 1.00 (0.87-1.16) | 1.48 (1.00-2.19) | 1.28 (0.80-2.05) | 0.94 (0.64-1.38) | 1.37 (1.25-1.50) |
| 100-999 | 2.10 (1.76-2.50) | 1.55 (1.22-1.96) | 0.96 (0.83-1.12) | 1.56 (1.06-2.30) | 1.08 (0.66-1.77) | 1.00 (0.68-1.47) | 1.52 (1.39-1.67) |
| 1,000-9,999 | 3.32 (2.82-3.89) | 1.75 (1.38-2.23) | 1.14 (0.97-1.33) | 1.31 (0.86-1.98) | 1.45 (0.90-2.36) | 1.02 (0.68-1.53) | 1.89 (1.72-2.07) |
| 10,000-99,999 | 4.34 (3.69-5.11) | 2.45 (1.93-3.10) | 1.13 (0.96-1.32) | 1.40 (0.92-2.12) | 0.81 (0.47-1.40) | 1.53 (1.07-2.21) | 2.13 (1.94-2.33) |
| ≥ 100,000 | 5.95 (4.96-7.14) | 2.58 (1.96-3.40) | 1.19 (1.00-1.42) | 1.77 (1.15-2.74) | 0.75 (0.40-1.40) | 2.49 (1.71-3.64) | 2.43 (2.19-2.69) |
| **Per Log10 cumulative HIV-1 RNA viral load unit increase (copy-years/mL)** | 1.43 (1.38-1.47) | 1.22 (1.16-1.27) | 1.04 (1.01-1.07) | 1.05 (0.98-1.13) | 0.93 (0.85-1.03) | 1.22 (1.14-1.30) | 1.18 (1.16-1.20) |

HPV: human papillomavirus

**Supplemental Table 10:** Hazard ratios and 95% confidence intervals quantifying the association between HIV-1 viremia and the incidence of specific infection-unrelated cancers and any infection-unrelated cancer. Models were adjusted for age, sex, calendar year, and CD4 cell count (lagged by six months), using different measures of HIV-1 viremia. All variables were time-updated. Individuals with no CD4 cell count measurement at or before the baseline visit were included in the study population.

|  | **Breast cancer** | **Colorectal cancer** | **Esophageal cancer** | **Leukemia** | **Lung cancer** | **Prostate cancer** | **Any infection-unrelated cancer** |
| --- | --- | --- | --- | --- | --- | --- | --- |
| **Per Log10 HIV-1 RNA viral load unit increase (copy/mL)** | 1.04 (0.99-1.08) | 1.11 (1.03-1.21) | 1.04 (0.95-1.14) | 1.29 (1.16-1.45) | 1.05 (0.98-1.14) | 0.98 (0.90-1.06) | 1.08 (1.05-1.10) |
| **Percentage time virally suppressed** |  |  |  |  |  |  |  |
| 80-100% (ref) | 1 | 1 | 1 | 1 | 1 | 1 | 1 |
| 50-79% | 0.97 (0.79-1.18) | 0.86 (0.54-1.37) | 1.50 (1.02-2.22) | 0.65 (0.28-1.54) | 1.21 (0.84-1.75) | 1.11 (0.79-1.58) | 1.12 (1.00-1.25) |
| 20-49% | 0.97 (0.74-1.27) | 1.10 (0.64-1.89) | 1.33 (0.78-2.25) | 0.91 (0.33-2.53) | 1.00 (0.60-1.68) | 1.15 (0.73-1.79) | 1.17 (1.01-1.35) |
| 0-19% | 1.07 (0.90-1.27) | 1.57 (1.17-2.12) | 1.20 (0.85-1.69) | 1.89 (1.25-2.85) | 1.25 (0.94-1.66) | 0.77 (0.55-1.08) | 1.30 (1.19-1.42) |
| **Cumulative HIV-1 RNA viral load (copy-years/mL)** |  |  |  |  |  |  |  |
| 0-9 (ref) | 1 | 1 | 1 | 1 | 1 | 1 | 1 |
| 10-99 | 1.03 (0.81-1.32) | 1.04 (0.65-1.65) | 0.88 (0.54-1.43) | 1.64 (0.86-3.13) | 0.90 (0.60-1.36) | 0.75 (0.48-1.16) | 1.04 (0.91-1.18) |
| 100-999 | 1.07 (0.84-1.36) | 1.26 (0.80-1.99) | 1.08 (0.67-1.75) | 2.21 (1.19-4.12) | 0.82 (0.54-1.25) | 0.80 (0.52-1.23) | 1.19 (1.04-1.35) |
| 1,000-9,999 | 1.13 (0.87-1.46) | 0.97 (0.58-1.64) | 1.11 (0.66-1.86) | 1.95 (0.99-3.83) | 0.94 (0.60-1.46) | 0.94 (0.59-1.49) | 1.29 (1.12-1.48) |
| 10,000-99,999 | 1.02 (0.78-1.33) | 1.64 (1.02-2.65) | 0.89 (0.52-1.52) | 2.29 (1.12-4.67) | 1.06 (0.69-1.63) | 0.71 (0.44-1.14) | 1.24 (1.07-1.42) |
| ≥ 100,000 | 1.08 (0.81-1.44) | 1.31 (0.75-2.27) | 1.34 (0.77-2.33) | 3.73 (1.72-8.09) | 1.01 (0.62-1.63) | 0.85 (0.52-1.39) | 1.24 (1.06-1.45) |
| **Per Log10 cumulative HIV-1 RNA viral load unit increase (copy-years/mL)** | 1.01 (0.96-1.06) | 1.08 (0.99-1.18) | 1.05 (0.95-1.15) | 1.22 (1.07-1.39) | 1.03 (0.95-1.11) | 0.99 (0.91-1.07) | 1.05 (1.02-1.07) |

**Supplemental Table 11:** Hazard ratios and 95% confidence intervals quantifying the association between HIV-1 viremia and the incidence of specific infection-related cancers and any infection-related cancer, using different measures of HIV-1 viremia. Models were adjusted for age, sex, calendar year, and CD4 cell count (lagged by six months). All variables were time-updated. Time-at-risk with no available 6-month lagged CD4 cell count was removed from the analysis.

|  | **Kaposi Sarcoma** | **Non-Hodgkin lymphoma** | **Cervical cancer** | **Other HPV-related**  **cancers** | **Hodgkin lymphoma** | **Conjunctival  cancer** | **Any infection-related cancer** |
| --- | --- | --- | --- | --- | --- | --- | --- |
| **Per Log10 HIV-1 RNA viral load unit increase (copy/mL)** | 1.39 (1.34-1.43) | 1.18 (1.13-1.25) | 1.04 (1.01-1.07) | 1.09 (1.02-1.17) | 0.96 (0.86-1.08) | 1.21 (1.13-1.29) | 1.16 (1.14-1.18) |
| **Percentage time virally suppressed** |  |  |  |  |  |  |  |
| 80-100% (ref) | 1 | 1 | 1 | 1 | 1 | 1 | 1 |
| 50-79% | 1.54 (1.21-1.97) | 1.59 (1.21-2.09) | 1.28 (1.12-1.47) | 0.92 (0.65-1.30) | 0.61 (0.33-1.12) | 1.50 (1.02-2.21) | 1.31 (1.19-1.44) |
| 20-49% | 2.10 (1.64-2.68) | 1.25 (0.87-1.78) | 1.25 (1.05-1.49) | 1.41 (0.97-2.04) | 0.74 (0.35-1.53) | 1.77 (1.18-2.67) | 1.39 (1.23-1.55) |
| 0-19% | 3.14 (2.77-3.58) | 1.48 (1.22-1.79) | 1.28 (1.13-1.43) | 1.32 (1.01-1.72) | 1.01 (0.67-1.53) | 2.02 (1.57-2.60) | 1.71 (1.60-1.83) |
| **Cumulative HIV-1 RNA viral load (copy-years/mL)** |  |  |  |  |  |  |  |
| 0-9 (ref) | 1 | 1 | 1 | 1 | 1 | 1 | 1 |
| 10-99 | 1.54 (1.21-1.95) | 1.22 (0.91-1.63) | 0.91 (0.76-1.08) | 1.40 (0.89-2.21) | 1.53 (0.83-2.81) | 0.88 (0.57-1.37) | 1.21 (1.09-1.36) |
| 100-999 | 1.84 (1.44-2.35) | 1.23 (0.90-1.67) | 0.88 (0.74-1.05) | 1.58 (1.01-2.47) | 1.22 (0.65-2.29) | 0.97 (0.62-1.51) | 1.27 (1.13-1.42) |
| 1,000-9,999 | 2.91 (2.31-3.68) | 1.39 (1.01-1.93) | 1.00 (0.83-1.21) | 1.37 (0.84-2.22) | 1.43 (0.75-2.75) | 0.96 (0.59-1.57) | 1.51 (1.34-1.70) |
| 10,000-99,999 | 4.18 (3.33-5.23) | 1.78 (1.31-2.42) | 1.07 (0.89-1.29) | 1.41 (0.87-2.29) | 0.73 (0.35-1.50) | 1.65 (1.08-2.54) | 1.78 (1.58-1.99) |
| ≥ 100,000 | 6.27 (4.93-7.97) | 2.27 (1.63-3.18) | 1.07 (0.87-1.32) | 1.79 (1.08-2.96) | 0.83 (0.39-1.81) | 2.55 (1.63-3.98) | 2.12 (1.87-2.40) |
| **Per Log10 cumulative HIV-1 RNA viral load unit increase (copy-years/mL)** | 1.44 (1.38-1.50) | 1.17 (1.10-1.23) | 1.04 (1.01-1.07) | 1.06 (0.98-1.14) | 0.89 (0.80-1.01) | 1.25 (1.16-1.35) | 1.16 (1.13-1.18) |

HPV: human papillomavirus

**Supplemental Table 12:** Hazard ratios and 95% confidence intervals quantifying the association between HIV-1 viremia and the incidence of specific infection-unrelated cancers and any infection-unrelated cancer, using different measures of HIV-1 viremia. Models were adjusted for age, sex, calendar year, and CD4 cell count (lagged by six months). All variables were time-updated. Time-at-risk with no available 6-month lagged CD4 cell count was removed from the analysis.

|  | **Breast cancer** | **Colorectal cancer** | **Esophageal cancer** | **Leukemia** | **Lung cancer** | **Prostate cancer** | **Any infection-unrelated cancer** |
| --- | --- | --- | --- | --- | --- | --- | --- |
| **Per Log10 HIV-1 RNA viral load unit increase (copy/mL)** | 1.01 (0.96-1.07) | 1.05 (0.95-1.16) | 1.03 (0.93-1.15) | 1.27 (1.10-1.47) | 1.09 (0.99-1.19) | 0.99 (0.90-1.08) | 1.05 (1.02-1.08) |
| **Percentage time virally suppressed** |  |  |  |  |  |  |  |
| 80-100% (ref) | 1 | 1 | 1 | 1 | 1 | 1 | 1 |
| 50-79% | 0.99 (0.80-1.23) | 0.77 (0.45-1.30) | 1.37 (0.89-2.11) | 0.49 (0.17-1.37) | 1.20 (0.80-1.81) | 0.99 (0.67-1.47) | 1.11 (0.98-1.25) |
| 20-49% | 0.94 (0.70-1.26) | 0.94 (0.51-1.76) | 1.15 (0.63-2.10) | 0.70 (0.21-2.28) | 0.94 (0.52-1.71) | 1.01 (0.60-1.70) | 1.07 (0.91-1.26) |
| 0-19% | 0.96 (0.78-1.18) | 1.35 (0.94-1.94) | 1.03 (0.68-1.55) | 1.63 (0.94-2.83) | 1.44 (1.03-2.02) | 0.86 (0.59-1.25) | 1.16 (1.04-1.29) |
| **Cumulative HIV-1 RNA viral load (copy-years/mL)** |  |  |  |  |  |  |  |
| 0-9 (ref) | 1 | 1 | 1 | 1 | 1 | 1 | 1 |
| 10-99 | 0.87 (0.66-1.16) | 1.03 (0.59-1.79) | 0.80 (0.45-1.44) | 1.73 (0.61-4.88) | 0.84 (0.50-1.40) | 0.85 (0.51-1.42) | 0.90 (0.77-1.05) |
| 100-999 | 0.90 (0.68-1.20) | 1.19 (0.69-2.07) | 0.94 (0.53-1.66) | 2.60 (0.95-7.12) | 0.70 (0.41-1.19) | 0.82 (0.49-1.37) | 0.98 (0.84-1.15) |
| 1,000-9,999 | 0.89 (0.66-1.21) | 0.87 (0.46-1.65) | 0.83 (0.44-1.58) | 2.19 (0.74-6.50) | 0.89 (0.51-1.56) | 1.00 (0.58-1.71) | 1.05 (0.89-1.24) |
| 10,000-99,999 | 0.84 (0.62-1.15) | 1.20 (0.66-2.18) | 0.71 (0.37-1.36) | 1.96 (0.64-6.04) | 1.13 (0.66-1.93) | 0.67 (0.38-1.19) | 0.99 (0.84-1.18) |
| ≥ 100,000 | 0.89 (0.63-1.24) | 1.21 (0.63-2.34) | 1.04 (0.54-2.02) | 3.33 (1.07-10.43) | 1.01 (0.56-1.83) | 0.89 (0.50-1.60) | 1.05 (0.87-1.26) |
| **Per Log10 cumulative HIV-1 RNA viral load unit increase (copy-years/mL)** | 0.99 (0.94-1.04) | 1.03 (0.93-1.14) | 1.00 (0.90-1.11) | 1.15 (0.97-1.36) | 1.06 (0.97-1.17) | 0.98 (0.89-1.07) | 1.02 (0.99-1.05) |

**Supplemental Table 13:** Hazard ratios and 95% confidence intervals quantifying the association between HIV-1 viremia and the incidence of specific infection-related cancers and any infection-related cancer, using different measures of HIV-1 viremia. Models were adjusted for age, sex, and calendar year. Individuals with CD4 cell counts <200 cells/µl at any time point were excluded from this analysis.

|  | **Kaposi Sarcoma** | **Non-Hodgkin lymphoma** | **Cervical cancer** | **Other HPV-related**  **cancers** | **Hodgkin lymphoma*** | **Conjunctival  cancer** | **Any infection-related cancer** |
| --- | --- | --- | --- | --- | --- | --- | --- |
| **Per Log10 HIV-1 RNA viral load unit increase (copy/mL)** | 1.61 (1.51-1.71) | 1.49 (1.37-1.62) | 1.12 (1.06-1.18) | 1.24 (1.09-1.42) | 1.06 (0.87-1.29) | 1.51 (1.28-1.79) | 1.32 (1.27-1.36) |
| **Percentage time virally suppressed** |  |  |  |  |  |  |  |
| 80-100% (ref) | 1 | 1 | 1 | 1 | 1 | 1 | 1 |
| 50-79% | 1.69 (0.91-3.16) | 1.20 (0.52-2.77) | 1.04 (0.73-1.47) | 0.95 (0.38-2.37) | 0.54 (0.13-2.26) | 0.46 (0.06-3.37) | 1.11 (0.86-1.43) |
| 20-49% | 2.90 (1.60-5.25) | 0.96 (0.30-3.04) | 1.29 (0.87-1.92) | 1.27 (0.46-3.49) |  | 3.23 (1.14-9.20) | 1.40 (1.05-1.87) |
| 0-19% | 4.15 (3.39-5.08) | 2.74 (2.05-3.65) | 1.61 (1.33-1.94) | 1.59 (1.00-2.53) | 1.53 (0.82-2.86) | 3.45 (1.99-5.97) | 2.36 (2.12-2.63) |
| **Cumulative HIV-1 RNA viral load (copy-years/mL)** |  |  |  |  |  |  |  |
| 0-9 (ref) | 1 | 1 | 1 | 1 | 1 | 1 | 1 |
| 10-99 | 1.69 (1.22-2.35) | 2.02 (1.32-3.10) | 1.10 (0.87-1.37) | 2.76 (1.50-5.05) | 1.95 (0.87-4.36) | 1.35 (0.57-3.22) | 1.59 (1.37-1.85) |
| 100-999 | 2.28 (1.64-3.17) | 2.58 (1.66-4.01) | 0.97 (0.76-1.23) | 2.45 (1.29-4.65) | 0.98 (0.38-2.53) | 1.56 (0.64-3.84) | 1.60 (1.37-1.88) |
| 1,000-9,999 | 4.47 (3.25-6.15) | 3.11 (1.91-5.06) | 1.17 (0.89-1.54) | 1.62 (0.73-3.59) | 2.13 (0.85-5.32) | 0.95 (0.28-3.19) | 2.12 (1.78-2.52) |
| 10,000-99,999 | 7.98 (5.70-11.17) | 5.80 (3.55-9.49) | 1.27 (0.93-1.72) | 2.27 (0.97-5.28) | 0.53 (0.11-2.57) | 3.62 (1.38-9.50) | 2.84 (2.35-3.41) |
| ≥ 100,000 | 13.29 (8.65-20.42) | 8.15 (4.24-15.67) | 1.26 (0.81-1.96) | 5.30 (2.15-13.02) | 1.36 (0.27-6.82) | 11.16 (4.08-30.56) | 3.90 (3.06-4.96) |
| **Per Log10 cumulative HIV-1 RNA viral load unit increase (copy-years/mL)** | 1.68 (1.57-1.80) | 1.48 (1.34-1.63) | 1.05 (0.99-1.11) | 1.15 (1.00-1.33) | 0.98 (0.78-1.22) | 1.50 (1.25-1.79) | 1.27 (1.22-1.31) |

HPV: human papillomavirus; *Due to categories with no events, the 20-49% and 50-79% categories were combined when estimating the association between percentage time virally suppressed and Hodgkin lymphoma

**Supplemental Table 14:** Hazard ratios and 95% confidence intervals quantifying the association between HIV-1 viremia and the incidence of specific infection-unrelated cancers and any infection-unrelated cancer, using different measures of HIV-1 viremia. Models were adjusted for age, sex, and calendar year. Individuals with CD4 cell counts <200 cells/µl at any time point were excluded from this analysis.

|  | **Breast cancer** | **Colorectal cancer** | **Esophageal cancer*** | **Leukemia*** | **Lung cancer** | **Prostate cancer** | **Any infection-unrelated cancer** |
| --- | --- | --- | --- | --- | --- | --- | --- |
| **Per Log10 HIV-1 RNA viral load unit increase (copy/mL)** | 1.10 (1.01-1.20) | 1.13 (0.97-1.33) | 0.93 (0.76-1.14) | 1.29 (1.05-1.58) | 1.22 (1.06-1.40) | 1.07 (0.91-1.25) | 1.20 (1.14-1.25) |
| **Percentage time virally suppressed** |  |  |  |  |  |  |  |
| 80-100% (ref) | 1 | 1 | 1 | 1 | 1 | 1 | 1 |
| 50-79% | 0.92 (0.55-1.52) | 1.65 (0.71-3.87) | 0.26 (0.04-1.87) | 1.63 (0.86-3.10) | 1.35 (0.54-3.40) | 0.92 (0.37-2.30) | 1.18 (0.89-1.56) |
| 20-49% | 0.60 (0.26-1.34) | 2.29 (0.92-5.74) | 0.46 (0.06-3.29) |  | 0.89 (0.22-3.66) | 1.29 (0.47-3.54) | 1.02 (0.70-1.51) |
| 0-19% | 1.54 (1.16-2.05) | 1.49 (0.85-2.59) | 0.81 (0.39-1.71) |  | 1.66 (1.02-2.69) | 0.89 (0.47-1.68) | 1.78 (1.53-2.07) |
| **Cumulative HIV-1 RNA viral load (copy-years/mL)** |  |  |  |  |  |  |  |
| 0-9 (ref) | 1 | 1 | 1 | 1 | 1 | 1 | 1 |
| 10-99 | 1.03 (0.72-1.47) | 1.26 (0.59-2.66) | 0.84 (0.41-1.74) | 1.23 (0.44-3.43) | 1.11 (0.61-2.01) | 0.63 (0.31-1.29) | 1.12 (0.91-1.36) |
| 100-999 | 0.96 (0.67-1.39) | 2.49 (1.23-5.05) | 0.90 (0.43-1.88) | 2.81 (1.13-7.02) | 1.15 (0.61-2.17) | 1.16 (0.59-2.24) | 1.44 (1.18-1.77) |
| 1,000-9,999 | 1.11 (0.73-1.68) | 1.69 (0.71-4.06) | 0.44 (0.16-1.25) | 1.72 (0.54-5.41) | 0.81 (0.36-1.86) | 1.08 (0.50-2.35) | 1.67 (1.33-2.09) |
| 10,000-99,999 | 0.98 (0.60-1.60) | 2.99 (1.29-6.95) | 0.50 (0.18-1.40) | 1.71 (0.42-6.96) | 1.96 (0.95-4.05) | 0.84 (0.34-2.07) | 1.61 (1.24-2.08) |
| ≥ 100,000 | 1.12 (0.58-2.17) | 3.32 (1.11-9.95) |  | 3.16 (0.60-16.62) | 1.37 (0.45-4.24) | 1.10 (0.38-3.23) | 1.54 (1.07-2.21) |
| **Per Log10 cumulative HIV-1 RNA viral load unit increase (copy-years/mL)** | 1.01 (0.92-1.11) | 1.27 (1.09-1.48) | 0.82 (0.66-1.02) | 1.20 (0.94-1.52) | 1.10 (0.94-1.28) | 1.06 (0.91-1.24) | 1.13 (1.08-1.18) |

*Due to categories with no events, the 50-79%, 20-49%, and 0%-19% categories were combined when estimating the association between percentage time virally suppressed and Leukemia, and the 10,000-99,999 and ≥ 100,000 categories were combined when estimating the association between cumulative HIV-1 RNA viral load and esophageal cancer.

**Supplemental Figure 1:** Flowchart showing the selection of the study population.


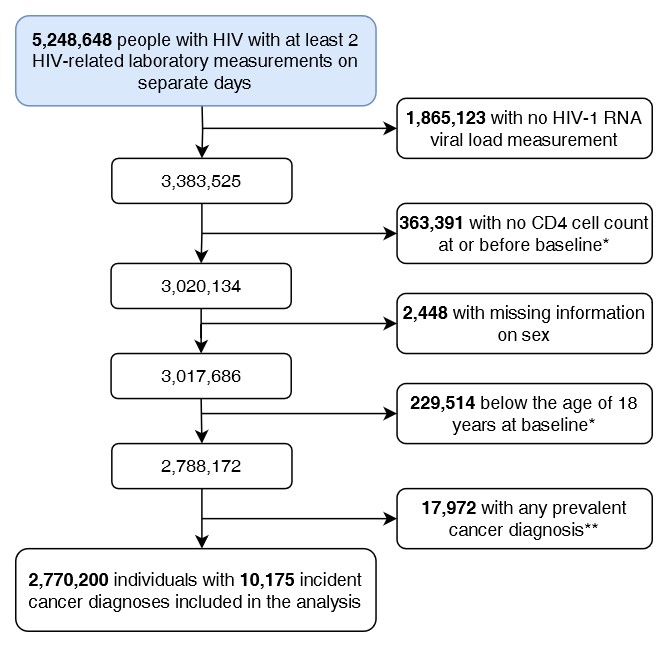


* baseline is the time of first HIV-1 RNA viral load measurement

**of any type; the number excluded varies depending on the cancer type of interest
